# A transcriptomic view to wounding response in young Scots pine stems

**DOI:** 10.1038/s41598-021-82848-3

**Published:** 2021-02-12

**Authors:** Kean-Jin Lim, Tanja Paasela, Anni Harju, Martti Venäläinen, Lars Paulin, Petri Auvinen, Katri Kärkkäinen, Teemu H. Teeri

**Affiliations:** 1 Department of Agricultural Sciences, Viikki Plant Science Centre, University of Helsinki, P.O. Box 27, 00014 Helsinki, Finland; 2 Natural Resources Institute Finland (Luke), Finlandiantie 18, 58450 Punkaharju, Finland; 3 Institute of Biotechnology, University of Helsinki, P.O. Box 56, 00014 Helsinki, Finland; 4 Natural Resources Institute Finland (Luke), University of Oulu, P.O. Box 413, 90014 Oulu, Finland; 5 State Key Laboratory of Subtropical Silviculture, Zhejiang A&F University, Lin’an District, Hangzhou, 311300 Zhejiang China

**Keywords:** Genetics, Molecular biology, Plant sciences

## Abstract

We studied the stress response of five-year-old Scots pine xylem to mechanical wounding using RNA sequencing. In general, we observed a bimodal response in pine xylem after wounding. Transcripts associated with water deficit stress, defence, and cell wall modification were induced at the earliest time point of three hours; at the same time, growth-related processes were down-regulated. A second temporal wave was triggered either at the middle and/or at the late time points (one and four days). Secondary metabolism, such as stilbene and lignan biosynthesis started one day after wounding. Scots pine synthesises the stilbenes pinosylvin and its monomethyl ether both as constitutive and induced defence compounds. Stilbene biosynthesis is induced by wounding, pathogens and UV stress, but is also developmentally regulated when heartwood is formed. Comparison of wounding responses to heartwood formation shows that many induced processes (in addition to stilbene biosynthesis) are similar and relate to defence or desiccation stress, but often specific transcripts are up-regulated in the developmental and wounding induced contexts. Pine resin biosynthesis was not induced in response to wounding, at least not during the first four days.

## Introduction

Angiosperm and gymnosperm trees must deal with a variety of environmental challenges, including attempts of invasion by herbivorous insects and pathogenic fungi^1,2^. Trees handle these challenges with preformed and induced defences. For example, in Scots pine (*Pinus sylvestris* L.) the thick bark that contains heavily lignified cells acts as a primary barrier against harmful intruders and is a preformed passive defence^3^. The phloem and xylem of pine contain toxic compounds such as resin acids in resin ducts and these serve as a second layer of preformed defence, if the primary barrier fails to stop the invasion^4^. The pine stilbenes, pinosylvin (PS) and pinosylvin monomethyl ether (PSME), are secondary metabolites that play a dual role in both preformed and induced defence. They are induced by herbivory, wounding and pathogen attack in, e.g., xylem and needles, but form constitutively under developmental control when the sapwood passes into heartwood and is filled with extractives. Stilbenes are derived from the phenylpropanoid pathway, starting from conversion of phenylalanine to cinnamic acid by phenylalanine ammonia lyase (PAL). Although pine stilbene synthase (STS) is well characterised, the enzyme that converts cinnamic acid to cinnamoyl-CoA, the substrate for STS, is not identified in pine, and the pinosylvin *O*-methyltransferase (PMT2) responsible for PSME formation was only recently identified correctly^5^. Regulatory proteins triggering stilbene biosynthesis in Scots pine are not known at all.

Harju and colleagues conducted a mechanical wounding study on stems of three years old Scots pine seedlings^6^. They showed that concentration of stilbenes, resin acids and lignans was increased in the xylem next to the wound. Three months after wounding, PS and PSME concentration in xylem had increased from levels below detection to 3.8 and 6.7 mg/g dry weight, respectively. Other experimental treatments leading to PS and PSME accumulation, and to transcriptional induction of the *STS* gene in pine, include ozone fumigation^7^, fungal elicitation^8^, elicitor induction^9^ and ultraviolet light (UV-C) irradiation^10^.

Resin acids (non-volatile diterpenoids) along with volatile monoterpenoids and sesquiterpenoids, are synthesised in resin duct epithelia during tree development, but are also induced in response to stress^11^. Chemical analysis of pine stems after wounding showed that, in addition to stilbenes, concentration of resin acids and lignans was highest in the area next to the wound^6^. Resin acids are indeed released from resin ducts in response to herbivory and mechanical wounding^6,12,13^. Once secreted, volatile terpenoids gradually evaporate from the wound areas while non-volatile diterpenoids polymerise and harden, eventually sealing the wound^4^.

Lignans are derived from the same monolignol subunits as lignin. They are dimers of monolignols and, unlike lignin, optically active. Stereospecific dimerization is guided by dirigent proteins (DIR) that as such do not have enzymatic activity^14–16^. Lignans have been reported to play important roles in plant defence as phytoalexins^15,17^. However, they are also developmentally regulated and in Scots pine lignans can be found in knotwood^18^.

Harju and colleagues^6^ measured extractives in stems of Scots pine seedlings in order to calculate genetic parameters of their induction. Half-sibs used in the experiment showed very high heritabilities (*h*^2^) for wound induced PS and lignan concentration (0.71 – 1.03). Heritability was also estimated between the wound induced extractives in the seedlings and the developmentally programmed amount of extractives in the heartwood of their adult mother trees. The relatively high offspring-parent heritability for PS of 0.31 suggests shared genetic control of developmentally regulated and induced biosynthesis of PS.

We used here the same experimental setup, now with five years old seedlings, to investigate how the transcriptome of young Scots pine xylem reacts to mechanical wounding in the time scale of few hours to few days. Our aim was to define genes involved in PS and PSME biosynthesis (in addition to *STS*), find candidate regulatory genes for the pathway and to characterise the wounding response in the young xylem (without a pathogen inoculum) in general terms. Many stress response studies have been conducted on either the bark or the phloem, or both, with a portion of xylem (sapwood) included^19–23^, but in all of these studies wounding was combined with a pathogen inoculum. Finally, we wanted to extract similarities and differences between the wound response and heartwood formation, both processes being characterised by stilbene biosynthesis in Scots pine. The high parent–offspring heritability between heartwood and wound induced stilbene biosynthesis brings up a hypothesis that there are genetic components, e.g. regulatory genes, that are shared between the wounding response and heartwood formation.

## Results

### Scots pine transcriptomes in wounded stems

Stress response to mechanical wounding in Scots pine xylem was investigated in a time course experiment using five-year-old seedlings. At this age, the seedlings do not yet contain any heartwood. We first followed the *STS* (key enzyme of pine stilbene pathway) expression of the wounded and unwounded stems (with bark and phloem removed) with semiquantitative RT-PCR (Supplementary Fig. S1). The expression of *STS* was noticed distinctly at 3 h (3H) after wounding, reached a steady level at 24 h (1D), showed the highest level at 96 h (4D) and then declined. We sequenced RNA of stem samples at these time points (control, 3H, 1D, and 4D from four half-sib families, sixteen libraries altogether) using the SOLiD platform. Differential expression analysis using *edgeR* showed that out of the total 77 326 tentative consensus sequences in the *Pinus* EST collection (The Gene Index Databases, 2014) 17 584 survived the filtering for very low or missing expression and a total of 4595 transcripts were differentially expressed (false discovery rate FDR < 0.05) in the stem (xylem) at 3H, 1D or 4D after wounding, compared to the control (0H) (Supplementary Tables S1 and S2). From each time point, we obtained 37–126 million paired-end reads, and the average mapping rate of all libraries to the 77 326 member *Pinus* EST collection was 88% (Supplementary Table S3). The *Pinus* EST collection is a combined collection of expressed sequence tags from different pine species and due to high sequence similarity between the species they have been assembled into ‘tentative consensus’ (TC) sequences as if they originated from a single taxon^24^. The nature of the *Pinus* EST collection was discussed in our earlier study of heartwood formation^25^. The main advantage in using the *Pinus* EST collection for mapping, instead of an assembly generated from our data, is that we can directly address the expression level of genes that are practically not expressed, such as the resin acid biosynthesis genes during heartwood formation^25^. For comparison, we also converted the SOLiD platform colour space reads to nucleotides, assembled them using *Trinity*^26^, and then mapped the reads to the annotated assembly. The average mapping rate to the *Trinity* assembly was 77% (Supplementary Table S3) and the obtained differentially expressed data was very similar to data using the *Pinus* EST collection as a reference. We present here results with *Pinus* EST collection as it further contains more annotated transcripts than the *Trinity* assembly and the contigs are longer. For a small number of target sequences, verification by real-time quantitative RT-PCR was carried out (Supplementary Table S4), showing comparable results for each tested gene.

### An overview of changes in biological functions after wounding

The 4595 differentially expressed TCs were subjected to gene set analysis (GSA) using the R package *PIANO*^27^ to shed light on changes of biological functions after wounding from the transcriptome point of view. The gene ontology (GO) biological function terms of up- and down-regulated transcripts at each time point were used for the analysis.

**Figure 1:**
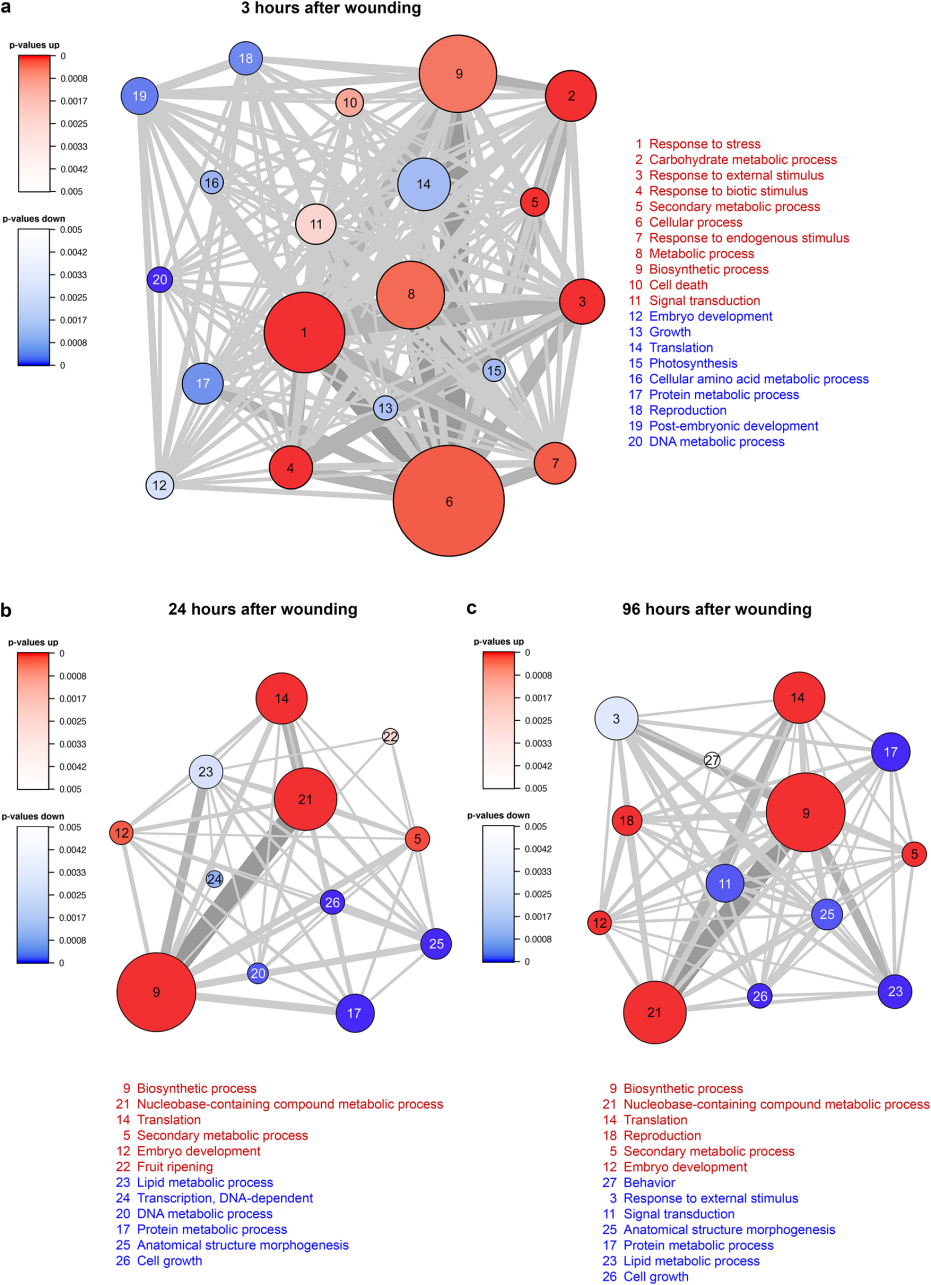
Gene set analysis of biological function changes at (**a**) 3 h, (**b**) 24 h, and (**c**) 96 h after wounding, compared to control. Biological function GO terms of statistically differentially expressed transcripts across all time points were given as input for the *PIANO*^27^ gene set analysis. The results were plotted on a distinct-directional network plot (with the gene set significance threshold at *p* < *0.005*). The size of each node represents the number of transcripts in the node. The width of the edges connecting the nodes indicates the number of shared transcripts between the nodes. The most significantly up-regulated (red) and down-regulated (blue) GO terms are on the top and the bottom of the list, respectively.

The GSA analysis showed that stress-related biological processes (such as ‘response to stress’, ‘response to biotic stimulus’, and ‘response to endogenous/external stimulus’) were up-regulated in the xylem at 3H after wounding (Fig. 1a). The corresponding induced transcripts were involved in water deficit, drought and dehydration stress^28–32^ or were defence-related^33–37^ (Supplementary Table S5). Concurrently, growth-related processes like ‘photosynthesis’, ‘growth’, ‘protein/DNA metabolic process’ were rapidly down-regulated (Fig. 1a). Many transcripts annotated with ‘carbohydrate metabolic process’ were up-regulated in the xylem 3H after wounding (Fig. 1a). These transcripts encode, for example, pectin methylesterase, glucanases and xyloglucan endotransglucosylases/hydrolases (XTHs; Supplementary Table S5)^38^, mainly associated with cell wall modification. Regulation of ‘secondary metabolism process’ (including lignin, lignan monomer and stilbene biosynthesis) was up-regulated in response to the drilling trauma (Fig. 1a,b). The transcripts activated 3H after wounding are likely to be of primary response genes, which can be induced after stimulation without de novo protein synthesis^39^.

One and four days after wounding GSA shows a very similar pattern, which differs dramatically from the 3H time point. Only the ‘biosynthetic’ and ‘secondary metabolic’ processes were up-regulated at all three time points. ‘Translation’ that was down-regulated at 3H, was up-regulated at 1D and 4D. Similarly, at one and four days after wounding, transcripts annotated with ‘embryo development’ were up-regulated (Fig. 1b,c). These transcripts encode proteins such as late embryogenesis abundant (LEA) proteins and chitinases. The growth-related processes ‘cell growth’ and ‘protein/DNA metabolic process’ remained down-regulated at these time points (Fig. 1b).

On the fourth day after wounding, expression of transcripts related to ‘response to external stimulus’ biological function were finally subsided. In contrast, transcripts annotated with the ‘reproduction’ GO term, down-regulated at 3H, were rebounded at this time point (Fig. 1c).

### Hierarchical clustering analysis

**Figure 2:**
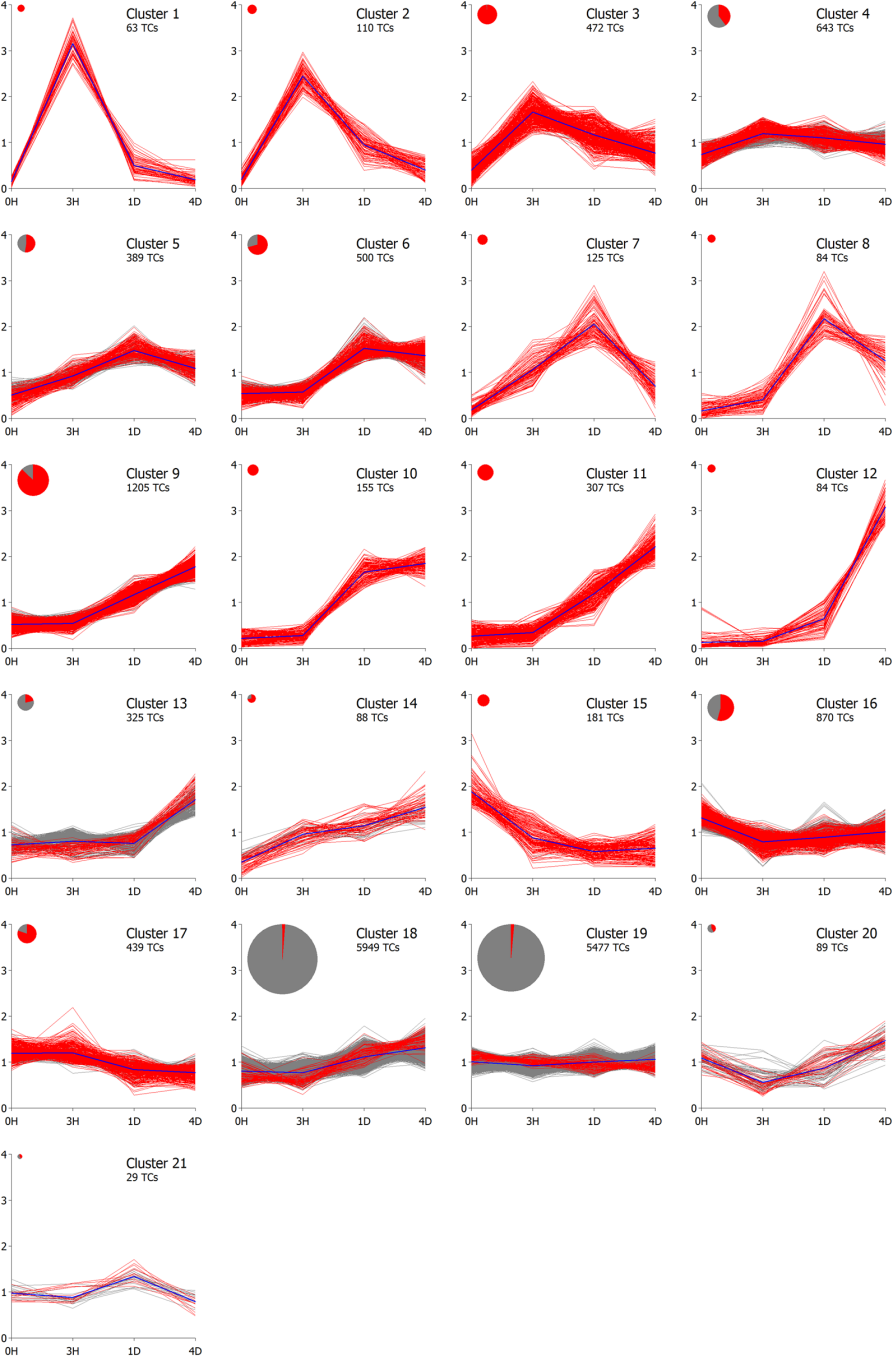
Expression profiles of transcripts in pine seedlings after wounding of the stem, grouped by the Bayesian hierarchical clustering algorithm *SplineCluster*^40^. All 17,584 *Pinus* EST transcripts that achieved at least eight CPM in at least four libraries are shown. The statistically differentially expressed transcripts (FDR < 0.05) at 3 h, 24 h or 96 h after wounding, compared to the control, are shown in red. The area of the disk shown for each cluster is proportional to the number of TCs in the cluster. 0H, control; 3H, 3 h; 1D, 24 h; 4D, 96 h.

In order to visualise the gene expression profiles in Scots pine seedling xylem in response to wounding, the total of 17 584 *Pinus* EST transcripts that achieved at least eight counts per million (CPM) in at least four libraries were subjected to hierarchical clustering analysis using the Bayesian hierarchical clustering algorithm *SplineCluster*^40^. Although *SplineCluster* distinguishes 21 clusters with the parameters used (Fig. 2, Supplementary Table S6), they can be sorted into only a few types of profiles. Clusters **1–4** (1288 TCs) show upregulation at 3H, which then returns to control levels during 1D and 4D. In clusters **1** and **2** (173 TCs) this pattern is very prominent. Another group of genes (clusters **5–8**; 1098 TCs) reacts more slowly and peaks at 1D, returning towards control at 4D. The third distinctive pattern (**9–14**; 2164 TCs) is shared by genes that are constantly up-regulated (compared to the control) until 4D, the actual peak of expression not visible in the experiment. One fifth of the differentially expressed genes showed a down regulation pattern during the time course (clusters **15–17**; 1490 TCs) and their expression does not recover during the time course. The two largest clusters are numbers **18** and **19** (11,426 TCs), showing a flat or slightly up-regulated pattern. In these clusters only 1.4% of the genes are differentially expressed, by the criteria we used (FDR < 0.05). Most of the other patterns are populated nearly exclusively by genes labelled differentially expressed by *edgeR*’s statistical analysis (Fig. 2).

Gene ontology (GO) enrichment analysis was carried out for differentially expressed TCs in each cluster and significantly enriched (FDR < 0.05) GO terms are listed in Supplementary Table S7, and summarised using *REVIGO*^41^ (Supplementary Fig. S2).

### Wounding triggers a bimodal stress response

In general, the five most dramatically enriched biological process GO terms, with FDR < 10^−100^, all relate to response to abiotic, biotic and endogenic stimulus, and to stress (and interestingly, to translation) (Supplementary Fig. S2 and Table S7). We observed that a number of plant defence related transcripts encoding proteins such as chitinases, defensin, pathogenesis-related (PR) proteins, pollen allergen CJP38, and a thaumatin-like protein^33–35^, were grouped in clusters **10–12** (Fig. 2, Supplementary Table S6) where the expression level was low at the early time points (0H and 3H), rose at 1D and continued to increase at 4D.

Other defence related transcripts, such as those encoding ENHANCED DISEASE SUSCEPTIBILITY 1 (EDS1), EDS1-like protein, PHYTOALEXIN DEFICIENT 4 (PAD4) and SENESCENCE ASSOCIATED GENE 101 (SAG101) were triggered already at 3H after wounding. Transcripts encoding nematode resistance protein HSPRO2, elicitor-responsive protein, and the whitefly induced protein Gp91-Phox, and a pleiotropic drug resistance protein were also triggered 3H after wounding. These early-triggered defence transcripts (grouped in cluster **3**) gradually decreased in their expression at 1D and 4D after wounding. To summarise, Scots pine triggered two temporal patterns of defence after wounding: a transient one at 3H and a second wave starting at 1D after wounding.

### Cell wall loosening is triggered as a strong pulse 3 h after wounding

Cluster **1** contains 63 TCs strongly up-regulated at 3H and returning back to control levels already at 1D after mechanical wounding. This interesting cluster enriches GO terms for the biological process of ‘carbohydrate metabolism’, molecular function of ‘transferase and hydrolase activity’ and the cellular compartment of ‘cell wall and extracellular region’ (Supplementary Table S7). The list of up-regulated genes (Supplementary Table S6) is populated by transcripts encoding XTHs and expansins^38,42^. Other expansins are induced later, in clusters **11** and **12**, along with transcripts encoding enzymes acting on cell wall polymers, such as mannan endo-1,4-beta-mannosidase, endo-beta-1,4-glucanase, glucan-1,3-beta-glucosidase and pectin methylesterase-like proteins^43–45^ (Supplementary Table S6).

### Dealing with the water loss caused by mechanical wounding

Transcripts encoding aquaporin, dehydrin, water deficit inducible protein LP3 and, in general, transcripts that were enriched with the GO term ‘response to abiotic stimulus’ were grouped in cluster **3** (Supplementary Table S7). They were triggered 3H after wounding, after which transcript levels gradually approached control levels during 1D and 4D. Transcripts encoding the EARLY RESPONSIVE TO DEHYDRATION (ERD) stress protein also grouped in cluster **3** (Supplementary Table S6). Other abiotic stimulus related transcripts, encoding cold acclimation protein, dehydrin, galactinol synthase, and LEA proteins, which are expressed during water deficit, drought and abiotic stress^30–32^ were grouped in cluster **7** and **8** (Supplementary Table S6) with a slower start but a similar recovery to control levels at 4D.

### Transcription factors induced in response to wounding

A number of plant transcription factors (TFs) were induced after wounding, some of them as early as 3H after treatment. In general, transcription levels of these TFs were resorted towards the control at late time points. Several transcription factors were moderately down-regulated after wounding and grouped in cluster **16** (Supplementary Table S6).

Of the differentially expressed TFs, plant WRKY TFs stand out (Supplementary Table S6). Some WRKY TFs were induced very rapidly in response to wounding and their expression resorted to control levels by the end of the experiment (clusters **1–3**). Others reacted more slowly and stayed up-regulated at 4D (clusters **5**, **6**, **10** and **14**). Still others showed moderate down regulation (cluster **16**).

Another prominent and up-regulated TF family is the NAC family. Typical to all differentially expressed NAC TFs was that their expression is reduced towards control levels after induction (clusters **3**, **4**,** 7** and **8**, Supplementary Table S6).

Up-regulated MYB TFs were found in clusters **8**,** 9** and **13**; two MYBs however were down-regulated and grouped in cluster **16**. Several basic-helix-loop TFs, in many cases acting as partners for MYB TFs, were also found up-regulated (clusters **3**,** 5** and **6**). Wounding of pine stem also induced transcription factors that belong to the APETALA 2/ethylene responsive factor (AP2/ERF) superfamily (clusters **6**, **7** and **15**, Supplementary Table S6).

### Secondary metabolism starts one day after wounding

Synthesis of stilbenes in pine is strongly induced by stress^6,8,9^. The signature enzyme of pine stilbene biosynthesis, stilbene synthase (STS), as well as pinosylvin *O*-methyltransferase 2 (PMT2) were induced 1D after wounding. The expression level of these transcripts continued to increase at the 4D time point (clusters **10** and **11**, Fig. 2, Supplementary Table S6). Transcripts for pinoresinol-lariciresinol reductase (PLR), a lignan biosynthetic enzyme that catalyses conversion of pinoresinol to lariciresinol and secoisolariciresinol^14^, were grouped in clusters **10** and **11**, sharing a similar expression profile as transcripts for STS and PMT2. The lignan pinoresinol is derived from coniferyl alcohol by single electron oxidation catalysed by peroxidases or laccases, and stereospecific dimerization by a dirigent protein^16^. The expression of transcripts encoding dirigent and dirigent-like protein increased to an average of 25–27 fold at 1D and 4D after wounding. These transcripts, together with pine stilbene biosynthetic transcripts and PLR were grouped in cluster **10** (Fig. 2).

Contrary to expectations, transcripts related to oleoresin biosynthesis, (-)-α-pinene synthase, diterpene synthase and abietadienol/abietadienal oxidase (CYP720B), were not correlated and showed different expression profiles in response to wounding. (-)-α-pinene synthase, responsible for biosynthesis of one of the volatile terpenes^46^, was grouped in cluster **12** (Fig. 2, Supplementary Table S6), and was not induced until 4D. CYP720B, responsible for biosynthesis of the resin acid abietic acid that is abundant in pine resin^13,47^, was down-regulated and grouped in cluster **15** (Fig. 2, Supplementary Table S6). Diterpene synthases were not differentially expressed in our experiment and transcripts related to resin biosynthesis were not triggered in response to wounding, at least not within four days.

### Comparison of heartwood formation and wounding stress transcriptomes

The pine stilbenes PS and PSME are synthesised both during heartwood formation and after wounding of stems. Strong induction of STS and PMT2 encoding transcripts can be observed in both cases, and in both cases drying of the tissue takes place. Comparison of the transcriptomes in the transition zone to heartwood^25^ and during wounding was done to uncover further shared gene activities in these processes. The RNA-seq libraries in our previous work on heartwood formation in Scots pine^25^ were mapped against the same *Pinus* EST collection, version 9.0^48^, with same criteria as the libraries in this study.

**Figure 3:**
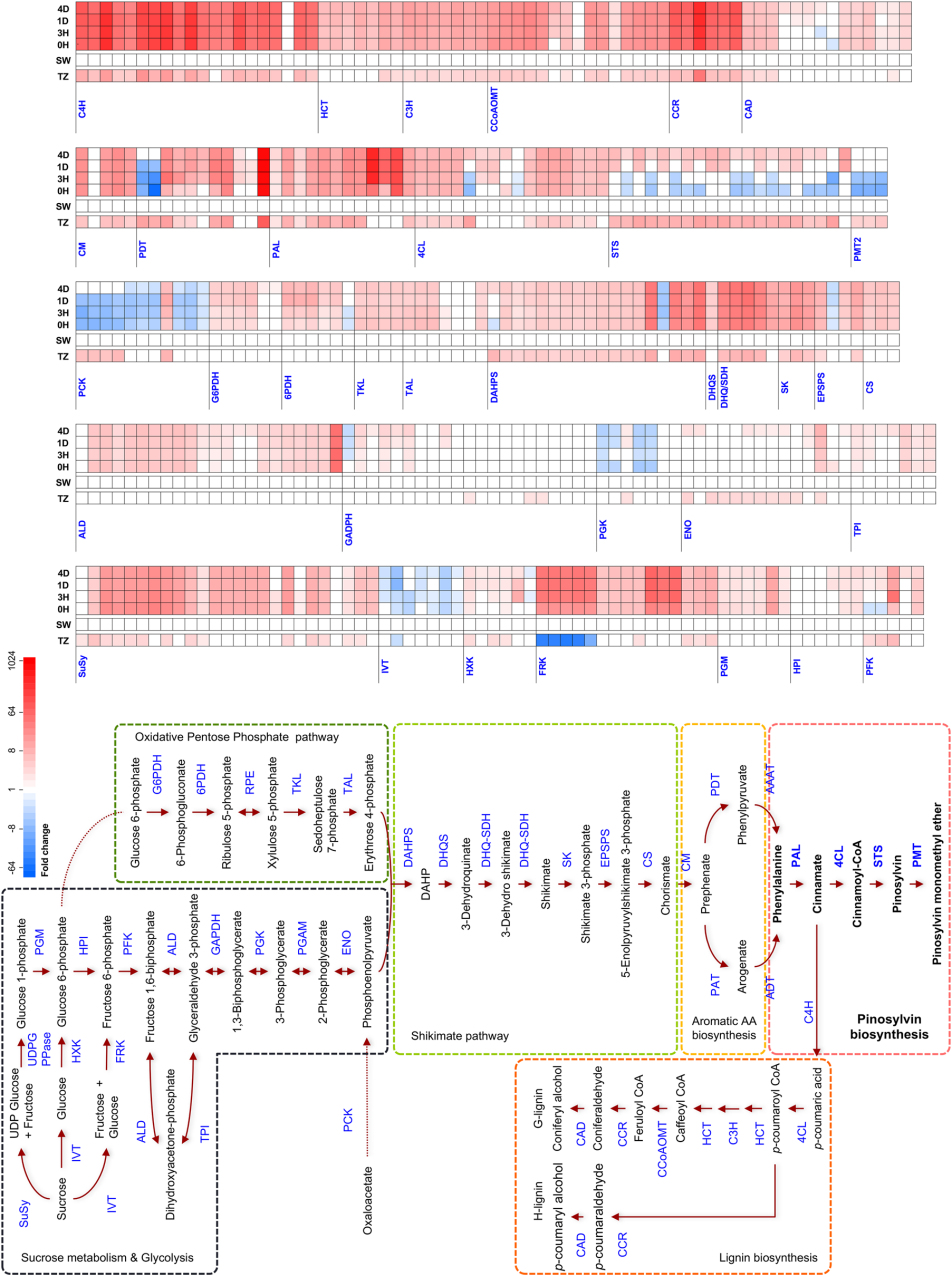
Comparison of the expressed transcripts between development and stress. RNA-seq library reads of the transition zone to heartwood, as well as 0H, 3H, 1D and 4D after mechanical wounding were all compared to the sapwood in a differential expression analysis with *edgeR*^78^. Fold change of each transcript, encoding enzymes from primary and secondary metabolic pathways to stilbenes and lignin monomers are presented as a heatmap, the colour indicating fold change for differential expression when it was statistically significant (FDR < 0.05). The enzymatic reactions in the pathways are represented with arrows and the enzymes are abbreviated in the following way: SuSy, sucrose synthase; IVT, invertase; UDPG PPase, UDP glucose pyrophosphorylase; HXK, hexokinase; FRK, fructokinase; PGM, phosphoglucomutase; HPI, hexose phosphate isomerase; PFK, phosphofructokinase; ALD, aldolase; GAPDH, glyceraldehyde 3-phosphate dehydrogenase; PGK, phosphoglycerate kinase; PGAM, phosphoglycerate mutase; ENO, enolase; TPI, triose phosphate isomerase; PCK, phosphoenolpyruvate carboxykinase; G6PDH, glucose 6-phosphate dehydrogenase; 6PDH, 6-phospogluconate dehydrogenase; RPE, ribulose 5-phosphate epimerase; TKL, transketolase; TAL, transladolase; DAHP, 3-Deoxy-D-arabinoheptulosonate-7-phosphate; DAHPS, DAHP synthase; DHQS, 3-dehydroquinate synthase; DHQ-SDH, dehydroquinate dehydratase-shikimate dehydrogenase; SK, shikimate kinase; EPSPS, 5-enolpyruvylshikimate 3-phosphate synthase; CS, chorismate synthase; CM, chorismate mutase; AA, amino acid; PAT, prephenate aminotransferase; ADT, arogenate dehydratase; PDT, prephenate dehydratase; AAAT, aromatic amino acid aminotransferase; PAL, phenylalanine ammonia lyase; 4CL, 4-coumarate:CoA ligase; STS, stilbene synthase; PMT, pinosylvin *O*-methyltransferase; C4H, trans-cinnamte 4-monooxygenase; HCT, hydroxycinnamoyl-CoA:shikimate/quinate hydroxycinnamoyltransferase; C3H, 4-coumaroyl shikimate/quinate 3′-hydroxylase; CCoAOMT, caffeoyl-CoA *O*-methyltransferase; CCR, cinnamoyl-CoA reductase; CAD, cinnamyl-alcohol dehydrogenase. 0H, control; 3H, 3 h; 1D, 24 h; 4D, 96 h; SW, sapwood; TZ, transition zone.

By comparing the differentially expressed transcripts between the wounding stress response and the transition zone transcriptomes, we found that a total of 424 TCs were coregulated in these conditions (Supplementary Table S8). In order to visualize this, we analysed the TCs encoding enzymes of these pathways for differential expression in a combined data set where all libraries were compared to the sapwood and to the heartwood transition zone transcriptomes (heartwood itself does not contain living cells) (Fig. 3).

## Discussion

### Balance between growth and defence

Through their lifetime, plants need to balance between growth and defence. Under biotic or abiotic stress, plants invest to the latter^49^. Already 3 h after wounding, stress-related biological processes were up-regulated in the xylem at the expense of growth related processes. On the fourth day after wounding the stress caused by wounding trauma was levelling off and transcripts related to e.g. reproduction were recovered.

Water deficit stress related transcripts were one of the early responding transcripts of Scots pine to mechanical wounding (drilling through) of the xylem. Strong induction of ERD, LEA, LP3 and dehydrin proteins was observed in the earliest time point (3 h). These proteins play a role in drought tolerance when the plant is under dehydration or cold stress^30,32,50^. Wang and colleagues^29^ hypothesised that the physiological role of LP3 was to protect the cell nucleus from water desiccation. Mechanical wounding causes evaporative water loss. Upregulation of these proteins may play a role to enhance cell survival under desiccation in wounded areas. Interestingly, we observed transcripts encoding desiccation-related proteins being up-regulated also in the transition zone of Scots pine^25^, during heartwood formation that includes drying of the wood. Nevertheless, these transcripts were not the same that were expressed in response to wounding, suggesting that Scots pine has two different sets of water deficit inducible proteins that respond specifically to development and wounding.

Besides dehydration stress-related genes, Scots pine responded to wounding with induction of genes encoding EDS1, PAD4, SAG101, elicitor-responsive and nematode resistance proteins at the earliest time point. EDS1 interacts with PAD4 and SAG101 to trigger defence against pathogen invasion and mediates the plant defence hormone salicylic acid biosynthesis^36,37,51^. A second temporal wave of defence was triggered at the middle and latest time points. These transcripts encoded antimicrobial peptides, chitinases, defensin, elicitor response proteins, pollen allergen, PR proteins, and thaumatin-like proteins. They have antimicrobial and antifungal activities and respond to elicitors and stresses caused by invasion of bacterial, insect, and fungal pathogens, or methyl jasmonate treatment and stresses such as cold^33–35,52–55^. Wounding by drilling through the stem (however without a concomitant pathogen inoculum) apparently mimicked a severe invasion of pathogens. Scots pine activated several defence pathways in order to encounter with potential invaders, as wounding exposed the inner xylem to the outside world.

The cell wall modification enzymes expansin, endoglucanase, and XTH are associated with modification of the cell wall by cleaving its hemicellulose-cellulose network to provide extensibility for growth^38,42,56,57^. Rapid induction of transcripts encoding these enzymes at early time points suggests that cell walls of wound areas started to undergo modification and remodelling very quickly.

The induction of lignan biosynthetic transcripts such as PLR, dirigent and dirigent-like protein at 1D and 4D after wounding implies that lignan deposition takes place in wounded areas. This is in accordance with our earlier study, where lignans were found accumulating in the pine xylem next to wounding^6^. Dirigent and dirigent-like proteins have been associated with response to abiotic/biotic stress and were induced also in spruce, in response to wounding and white pine weevil or western spruce budworm herbivory^58,59^. Besides lignans, pine stilbenes were synthesised in the xylem next to the wound^6^. We also observed that pine stilbene biosynthetic transcripts were induced in xylem at 1D and 4D as a response to wounding stress. Altogether, it can be concluded that pine was remodelling the cell walls at wound areas in order to maintain cell wall integrity, prevent invasion of pathogens, enhance mechanical strength for turgor pressure and to prevent further water loss.

Pine resin acids can be found in the bark, sapwood and heartwood. They act as defence barriers against herbivore invasion, particularly in the bark^12^. It was somewhat surprising that transcripts involved in resin acid biosynthesis and its upstream pathways were not induced in the xylem as a response to wounding in our data. Harju and colleagues^6^ observed that high amount of resin acids accumulated next to the wounded areas when they carried out a chemical analysis three months after wounding. It is possible that resin acid biosynthesis starts later after wounding, or that accumulation of resin acids in the wounded areas is exclusively due to transfer of resin through resin ducts.

One MYB and one NAC transcription factor was up-regulated during heartwood formation and was highly correlated to phenylpropanoid biosynthesis in our recent study^25^. These two transcription factors, however, were not induced in response to wounding. MYB TFs, like NAC TFs, are involved in secondary cell wall formation and are common regulators of plant secondary metabolism^25,60,61^, often in response to biotic stresses^62^. MYB TFs were reported regulating poplar (*Populus tremuloides*) secondary metabolites in response to abiotic (wounding and UV-B irradiation) and biotic (pathogen) stresses^62^. NAC transcription factors have roles in secondary cell wall formation^60^ but they are also involved in response to insect herbivores, fungal infection, wounding, cold and heat shock and dehydration^63–65^. In the results reported here, in addition to MYB and NAC, a wide array of TFs such as AP2/ERF, AP2, bHLH, DREB, ERF, MYC and WRKY was strongly induced either at 3H or 1D after wounding. In general, their expression level was reduced at 4D. AP2/ERF transcription factors have been reported to play a role in regulating plant responses to pathogen attack, wounding, abiotic stresses like cold, salinity and drought^66,67^. Members of the large WRKY TF family have been associated with plant defence^68,69^ and have been reported to play roles in response to biotic and abiotic stresses^70–72^.

### Wounding stress and heartwood formation transcriptomes converge in stilbene biosynthesis

Wounding caused by drilling of young stems and heartwood formation in adult trees share desiccation stress. Both during heartwood formation and after wounding of stems the pine stilbenes PS and PSME are synthesised and strong induction of STS and PMT2 encoding transcripts can be observed in both cases. As developmentally controlled and wound induced stilbene biosynthesis are genetically connected^6^, comparison of the transcriptomes in the transition zone to heartwood^25^ and during wounding (data presented here) may uncover further shared gene activities in these processes. By comparing the differentially expressed transcripts between the wounding stress response and the transition zone transcriptomes, we found that a total of 424 TCs were coregulated in both conditions (Supplementary Table S8).

Ethylene has been suggested to play an important role in both heartwood formation and in response to stress^73,74^. Ethylene biosynthesis related transcripts encoding 1-aminocyclopropane-1-carboxylate oxidase were up-regulated during both heartwood formation and in response to wounding (cluster **11**, Fig. 2). Auxin responsive genes, on the other hand, were down-regulated in both conditions (Supplementary Table S6).

Transcripts involved in plant defence such as Avr9 elicitor response protein, chitinases, and PR proteins were up-regulated in both conditions (Supplementary Table S8). Most of these defence-associated transcripts were grouped in cluster **10, 11** and **12** in the wounding transcriptomes (Fig. 2) and had an average of ninefold induction in the transition zone^25^. Defence-related transcripts, e.g. for chitinases or PR proteins, have been reported to be involved not only in response to stresses but also in different developmental contexts, including heartwood formation^52,75,76^.

We also observed that the aquaporin transcripts were differentially expressed in both conditions (Supplementary Table S8). Aquaporin transcripts were down-regulated in the heartwood formation transcriptome; however, they were up-regulated at early time points after wounding, then gradually reduced and clustered in cluster **3** (Fig. 2, Supplementary Table S6).

During heartwood formation we saw prominent upregulation in the transition zone of not only the stilbene biosynthesis pathway (STS and PMT2), but also its upstream pathways of phenylalanine biosynthesis (the shikimate pathway) and sugar metabolism, all the way to sucrose synthase, a general marker for metabolic activity^73^. In response to wounding, these upstream pathways are conspicuously absent in the lists of differentially expressed genes.

An obvious explanation is that these pathways are already active in the unwounded xylem of the five-year-old Scots pine seedlings, which were active in growth and lignin biosynthesis when the experiment started. Indeed, the upstream pathways for stilbene (and lignin) biosynthesis are already active in the unwounded stems and in most cases, are not up-regulated further (Fig. 3).

The exceptions to this pattern are interesting. For the entry point activity (PAL) of the phenylpropanoid pathway (including stilbene, lignin and lignan biosynthesis), the transcripts that were induced during heartwood formation are constitutively active in the wounding experiment. Interestingly, in addition, we can observe PAL transcripts not active in the transition zone but either constitutively active in the young stems or induced by mechanical wounding. A similar pattern is observed for 4CL transcripts, and for many others the gene expressed constitutively in the wounding experiment is not the one that is induced in heartwood formation (Fig. 3).

During heartwood formation, two transcription factors showed upregulation in the transition zone, a member of the MYB and the NAC family. TCs encoding these proteins were not up-regulated during wounding, instead other MYB and NAC family transcription factors were (Supplementary Table S6). It seems that the shared pathways of heartwood formation and wound response, in respect to stilbene biosynthesis, do not converge until at the level of genes that code for the respective enzymes.

## Conclusions

From these observations, we can conclude that Scots pine has a different set of MYB and NAC TFs that are expressed in response to stress and development. Similar division of tasks is seen also for the stress induced defence genes, and for the early steps of the phenylpropanoid pathway. As far as our resolution can tell, this does not apply to STS and PMT, unless very closely related paralogues of these loci have, after all, different duties in Scots pine. In fact, for the genetic components that link heartwood stilbene content of the mother trees to wound induced stilbene content of the seedlings^6^, our data are pointing directly to the structural genes encoding STS and PMT2.

## Methods

### Plant material, growth conditions and the mechanical wounding process

Seeds from four unrelated mother-trees were collected in February 2005 from a progeny trial of Scots pine located in Leppävirta (62°25′ N and 27°45′ E), eastern Finland. The trees were 39 years old during seed collection. Seeds from each mother-tree comprise a half-sib family (coded as T395, T413, T426 and T547), in which the fathers originate from an unknown stochastic pollen pool. The seeds were sown into plastic trays in May 2005 and were grown in a greenhouse at the Punkaharju Research Unit (61°48′ N and 29°19′ E, former Metla, currently Luke), Finland. During five years of growth, a sufficient level of irrigation, a low level of fertilization and no artificial illumination was applied. Aphids were controlled with pesticide (0.3% malathion). The mechanical wounding experiment was performed in May 2010 at the beginning of the sixth growing season. Ten 2.5 mm diameter holes with 1 cm interval were drilled through the stem on the fourth internode counted from the top of the seedling. Initial test sampling was carried out 0 (control), 3, 6, 12, 24, 48, 96 and 192 h after wounding. For RNA sequencing, three seedlings from each of the four families were treated and sampled 3, 24 and 96 h after wounding, respectively, and one as a control. Wounded stem sections and the corresponding section of the unwounded control were cut apart, and their bark and phloem were removed. The harvested stem sections were cut into pieces and frozen in liquid nitrogen immediately. The samples were kept at -80 °C before RNA extraction.

### Total RNA isolation, semiquantitative *STS* RT-PCR, qPCR and sample preparation for RNA sequencing

The frozen stem samples were further cut into smaller pieces and ground to fine powder using a milling machine (Qscillating Mill MM400 Retsch GmbH, Germany) with intermittent cooling with liquid nitrogen. Total RNA isolation, genomic DNA digestion, purification, RNA quantitation, and semiquantitative STS RT-PCR with first strand cDNA that was synthesised from a total of 500 ng of total RNA as template was performed as described in our earlier study^25^. For real-time quantitative RT-PCR (qPCR), cDNAs of each time point were pooled and qPCR was performed as described and with the primers described in our earlier study^25^. Sample preparation for RNA-seq for control and 3, 24 and 96 h after wounding was carried out as described earlier^25^.

### Mapping of RNA-seq libraries, data handling, and differential expression analysis

Paired-end colour space reads of control, 3, 24, and 96 h after wounding RNA-seq libraries were mapped against the *Pinus* EST collection, version 9.0 obtained from Pine Gene Index^48^ using the *SHRiMP2*^77^ alignment tool. Mapping parameters and data handling after mapping were performed as described earlier^25^. The computational tasks were carried out on the Finnish Grid Infrastructure clusters and supercluster server in CSC- IT Center for Science Ltd, Espoo, Finland. Table of mapped read counts for the RNA-seq libraries was imported into an *edgeR* R session^78^. The low expression transcripts of *Pinus* ESTs collection were filtered, and only TCs that achieved at least eight counts per million (CPM) in at least four libraries were kept. Differential expression analysis was carried out as described before^25^.

Some of the *Pinus* EST collection annotations are either missing or not informative. We updated the annotations as described in our previous study^25^. The original and updated annotations are listed in Supplementary Table S2.

### De novo assembly and differential expression analysis

Paired-end colour space reads of each RNA-seq library were converted to nucleotides with an in-house tool. Next, de novo assembly was performed using the *Trinity* software^26^ with default assembly parameters, except that *SS_lib_type* (library type parameter) and *min_kmer_cov* (minimum kmer threshold abundance) was set to FR and 2, respectively. The annotations of the *Trinity* assembly were obtained using the blastx algorithm^79^. The converted colour space reads for each time point were then mapped against the *Trinity* assembly. Data handling and differential expression analysis were performed as described above. The RNA-seq reads have been submitted to NCBI under Bioproject PRJNA631777.

### Bayesian hierarchical clustering analysis

The 17 584 *Pinus* EST transcripts (including the 4595 TCs that were differentially expressed across some time points) that achieved at least eight CPM mapped counts were clustered using *SplineCluster*, a Bayesian model-based hierarchical clustering algorithm for time series data^40^. The average CPM counts of the four libraries at each time point for each transcript were first calculated. These mean CPM counts were divided by the average CPM counts across all time points. With this normalisation, the average expression level of each gene is set to 1 and the expression level can vary between 0 and 4, however the fold change between time points remains unchanged. Next, Bayesian hierarchical clustering analysis was carried out with default parameters except *priorprecision* and *normalisetargets* was set to 1e-30 and 0, respectively. Visualisation of the clusters was done using an in-house tool that used the *SplineCluster* data as input.

### Gene ontology enrichment analysis

Gene Ontology (GO) terms of *Pinus* EST collection was obtained and complexity of obtained GO terms was reduced using Blast2GO^80^. GO enrichment analysis for differentially expressed transcripts in each cluster was performed as before^25^. Enriched GO lists were summarised and visualised in semantic similarity-based scatterplots using *REVIGO*^41^ with the *allowed similarity* parameter set to 0.5 and other parameters as default.

### Gene set analysis

Gene set analysis (GSA) was performed with the *PIANO* R package^27^. The logarithm of fold change value, the p-value and the GO terms for 4595 differentially expressed transcripts across all time points were used as input in a *PIANO* R session. The gene set analysis was carried out with default parameters. The GSA results were visualised in a gene set network plot in distinct-directional class with significance level at p < 0.005.

## Supplementary Information

Supplementary Figure.

Supplementary Table 1.

Supplementary Table 2.

Supplementary Table 3.

Supplementary Table 4.

Supplementary Table 5.

Supplementary Table 6.

Supplementary Table 7.

Supplementary Table 8.
