# Supplementary material for: A transcriptomic view to wounding response in young Scots pine stems
Source: Sci Rep. 2021 Feb 12;11:3778. doi: 10.1038/s41598-021-82848-3 (PMC7881122; doi:10.1038/s41598-021-82848-3)
Supplement: Supplementary file 1 — Supplementary Figure. [file 41598_2021_82848_MOESM1_ESM.pdf]

**A transcriptomic view to wounding response in young Scots pine stems**

Kean-Jin Lim, Tanja Paasela, Anni Harju, Martti Venäläinen, Lars Paulin, Petri Auvinen, Katri Kärkkäinen and Teemu H. Teeri

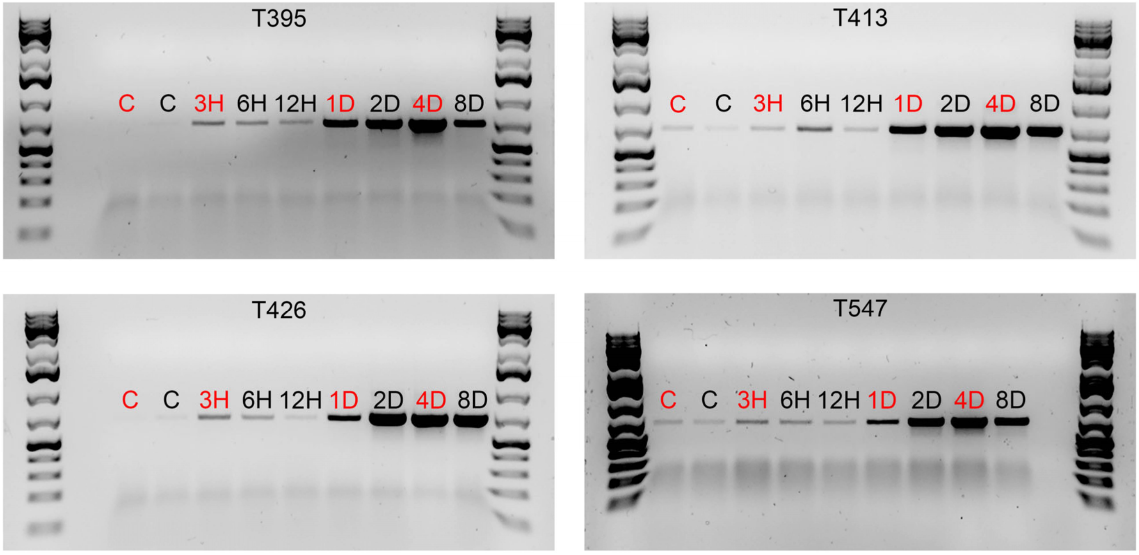


**Supplementary Figure S1.** Semiquantitative RT- PCR of STS transcript (853 bp band) of the wounded and unwounded stems (bark and phloem removed) at different time points. The expression of STS showed a distinct level at 3 hours (3H), reached a stable level at 24 hours (1D), and showed the strongest level at 96 hours (4D) after wounding. Wounded stems of these time points (3H, 1D, 4D) together with unwounded stem (control, C) were sequenced (marked in red). C, control; 3H, 3 hours; 6H, 6 hours; 12H, 12 hours; 1D, 24 hours; 2D, 48 hours; 4D, 96 hours; 8D, 192 hours.The size marker is GeneRuler 1 kb DNA Ladder
(Thermo Fischer Scientific).

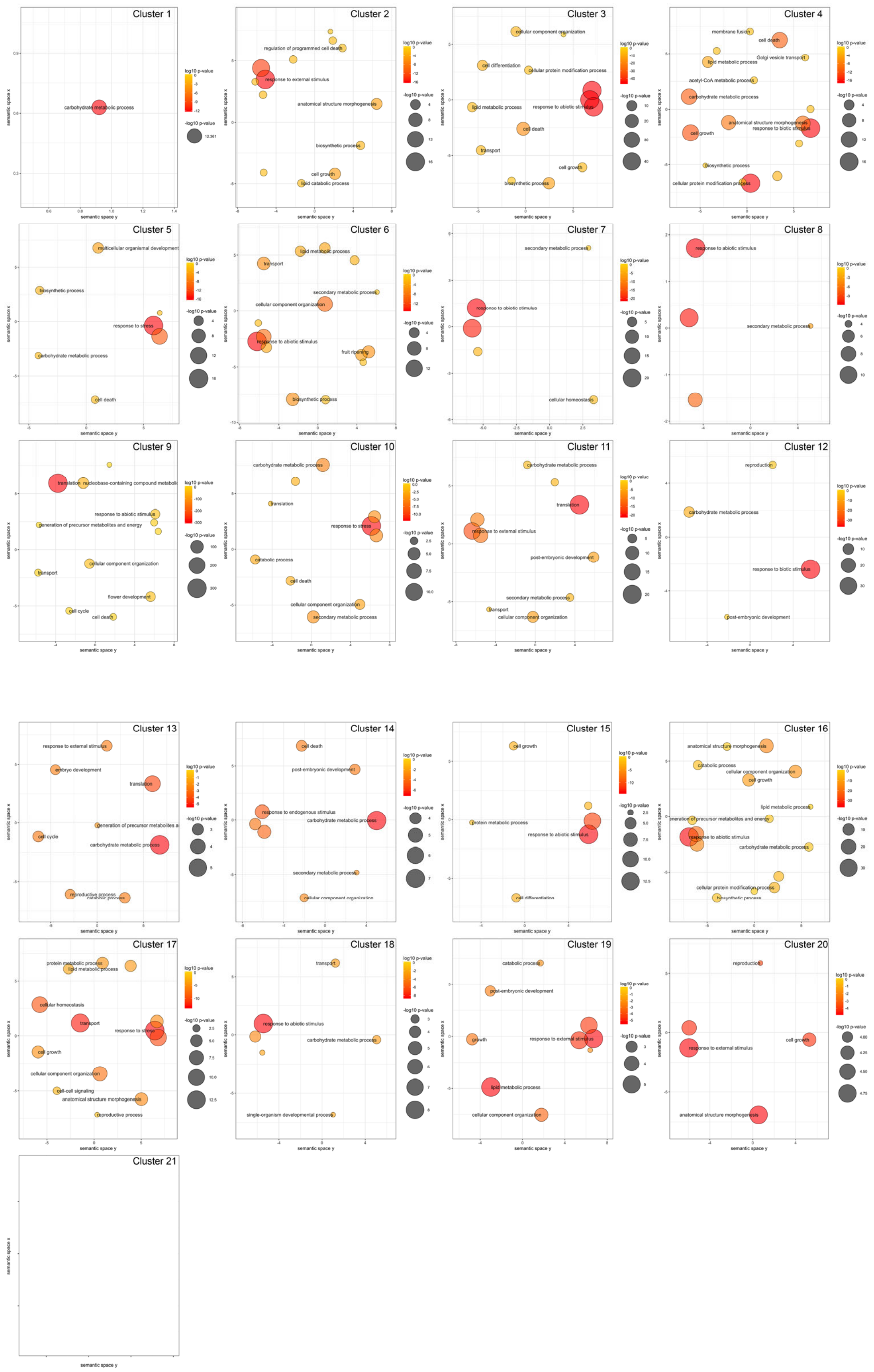


**Supplementary Figure S2.** Gene Ontology (GO) enrichment analysis for each cluster with redundant GO terms reduced and summarised using the *REVIGO* algorithm[41]. Representative GO terms (having the most significant p-value in the cluster) for each cluster are shown in a two-dimensional scatter plot, where GO terms with semantic similarity group close to each other (on an arbitrary scale). The node colour and size indicates the p-value derived from the GO enrichment analysis.
